# Effects of beta-alanine supplementation on body composition: a GRADE-assessed systematic review and meta-analysis

**DOI:** 10.1080/15502783.2022.2079384

**Published:** 2022-05-31

**Authors:** Damoon Ashtary-Larky, Reza Bagheri, Matin Ghanavati, Omid Asbaghi, Alexei Wong, Jeffrey R. Stout, Katsuhiko Suzuki

**Affiliations:** a Ahvaz Jundishapur University of Medical Sciences, Nutrition and Metabolic Diseases Research Center, Ahvaz, Iran; b Department of Exercise Physiology, University of Isfahan, Isfahan, Iran; c Shahid Beheshti University of Medical Sciences, National Nutrition and Food Technology Research Institute, Faculty of Nutrition Sciences and Food Technology, Teheran, Iran; d Shahid Beheshti University of Medical Sciences, Cancer Research Center, Tehran, Iran; e Marymount University, Department of Health and Human Performance, Arlington, VA, USA; f University of Central Florida, Institute of Exercise Physiology and Physical Therapy, School of Kinesiology and Physical Therapy, Orlando, FL, USA; g Waseda University, Faculty of Sport Sciences, Tokorozawa, Japan

**Keywords:** beta-alanine, body composition, meta-analysis, systematic review

## Abstract

**Purpose:**

Previous studies have suggested that beta-alanine supplementation may benefit exercise performance, but current evidence regarding its effects on body composition remains unclear. This systematic review and meta-analysis aimed to investigate the effects of beta-alanine supplementation on body composition indices.

**Methods:**

Online databases, including PubMed/Medline, Scopus, Web of Science, and Embase, were searched up to April 2021 to retrieve randomized controlled trials (RCTs), which examined the effect of beta-alanine supplementation on body composition indices. Meta-analyses were carried out using a random-effects model. The I^2^ index was used to assess the heterogeneity of RCTs.

**Results:**

Among the initial 1413 studies that were identified from electronic databases search, 20 studies involving 492 participants were eligible. Pooled effect size from 20 studies indicated that beta-alanine supplementation has no effect on body mass (WMD: −0.15 kg; 95% CI: −0.78 to 0.47; *p* = 0.631, I^2^ = 0.0%, *p* = 0.998), fat mass (FM) (WMD: −0.24 kg; 95% CI: −1.16 to 0.68; *p* = 0.612, I^2^ = 0.0%, *p* = 0.969), body fat percentage (BFP) (WMD: −0.06%; 95% CI: −0.53 to 0.40; *p* = 0.782, I^2^ = 0.0%, *p* = 0.936), and fat-free mass (FFM) (WMD: 0.05 kg; 95% CI: −0.71 to 0.82; *p* = 0.889, I^2^ = 0.0%, *p* = 0.912). Subgroup analyses based on exercise type (resistance training [RT], endurance training [ET], and combined training [CT]), study duration (<8 and ≥8 weeks), and beta-alanine dosage (<6 and ≥6 g/d) demonstrated similar results. Certainty of evidence across outcomes ranged from low to moderate.

**Conclusions:**

This meta-analysis study suggests that beta-alanine supplementation is unlikely to improve body composition indices regardless of supplementation dosage and its combination with exercise training. No studies have examined the effect of beta-alanine combined with both diet and exercise on body composition changes as the primary variable. Therefore, future studies examining the effect of the combination of beta-alanine supplementation with a hypocaloric diet and exercise programs are warranted.

## 1. Introduction

Various nutritional strategies are recommended to improve body composition by decreasing body fatness (both fat mass [FM] and body fat percentage [BFP]) and/or enhancing lean mass [1–3]. The use of protein sources, often combined with exercise training to improve body composition, is prevalent among both athletes and the general population [1]. Indeed, the beneficial effects of protein-rich foods, such as egg [1], milk [3], soy [2], and meat [4] on FM loss and lean mass gains are well established. Non-protein compounds are also used to improve body composition as evidence suggests they play important physiological roles, such as metabolic intermediates, biomolecular components, and post-translational modifiers [5].

Beta-alanine, in particular, has gained considerable interest for this purpose and provides the focus of this investigation. Beta-alanine, a non-proteogenic amino acid, has become an increasingly popular dietary supplement as it boosts intramuscular carnosine (beta-alanyl-L-histidine) concentrations, which augments the fatigue threshold and improves high-intensity exercise performance [6]. This beneficial advantage of beta-alanine has increased its utilization among athletes. In this regard, a systematic review of 19 randomized controlled trials (RCTs) showed that beta-alanine supplementation increases athletic performance [7]. In another review study, its beneficial effects on exercise homeostasis and excitation-contraction coupling have also been indicated [6]. Taken together, most of the literature has focused on beta-alanine’s effects on exercise performance [6–11]. However, its effects on body composition are less studied. It has been hypothesized that beta-alanine supplementation could lead to improvements in lean mass by increasing the volume of training, although evidence is equivocal. For instance, beta-alanine supplementation increased lean mass after 3 weeks of high-intensity interval training (HIIT) in recreationally active college-aged men [11]. On the other hand, Kern et al. did not report changes in body composition or lean mass after beta-alanine supplementation for 8 weeks in previously trained athletes [12]. Additionally, 28 days of beta-alanine supplementation failed to affect body composition in female master athletes [13]. Likewise, no significant effects of 10 weeks of resistance training combined with beta-alanine supplementation were observed on BFP [14]. These conflicting outcomes indicate a need to conduct a systematic review and meta-analysis to assess the effects of beta-alanine supplementation on this topic. Therefore, we conducted a systematic review and meta-analysis to investigate beta-alanine’s effects on body composition indices (body mass, BFP, FM, and fat-free mass [FFM]).

## 2. Methods

This study was performed based on the Preferred Reporting Items for Systematic Reviews and Meta-Analyses (PRISMA) protocol to conduct and disseminate systematic reviews and meta-analyses [15].

## 3. Search strategy

To find interrelated studies on beta-alanine supplementation in adults, we performed a comprehensive literature search in online databases including PubMed/Medline, Scopus, Web of Science, and Embase for the time period up to April 2021. The following terminology was utilized in the search: (“β-alanine’ OR ‘beta-alanine’ OR ‘b-alanine supplementation’ OR ‘beta-alanine supplementation’ OR ‘beta alanine’ OR ‘carnosine’ OR ‘βalanine’ and ‘beta-alanine’) AND (‘Intervention Study’ OR ‘Intervention Studies’ OR ‘controlled trial’ OR randomized OR randomized OR random OR randomly OR placebo OR ‘clinical trial’ OR ‘randomized controlled trial’ OR ‘randomized clinical trial’ OR RCT OR blinded OR ‘double blind’ OR ‘double blinded’ OR ‘clinical trial’ OR trials OR ‘Pragmatic Clinical Trial’ OR ‘Cross-Over Studies’ OR ‘Cross-Over’ OR ‘Cross-Over Study’ OR parallel OR ‘parallel study’ OR ‘parallel trial’). Search parameters were not restricted to publication date or original printed language. References from all relevant peer-reviewed investigations were consulted and cross-referenced against database searches to avoid omitting publications. All citations were subsequently included in the Endnote screening software, and duplicates were later removed from consideration in this study.

## 4. Inclusion criteria

In the present study, consideration was given to studies meeting all of the PICO criteria: (Participants) Adults (subjects older than 18 years), (Intervention) used a beta-alanine supplementation intervention/regimen, (Comparison) included a placebo or control group, (outcomes) body composition variables as an outcome (body mass, BFP, FM, and FFM). In the event of multiple cohort data publications from a single larger dataset, the more comprehensive article, whenever possible, was utilized in the present study. Studies containing more than one intervention group meeting the above criteria were considered independent datasets to determine the overall effect size.

## 5. Exclusion criteria

Investigation excluded from consideration comprised [1]: cross-sectional or case-control design [2], non-RCTs and literature reviews [3], ecological studies [4], control group manipulation of any sort [5], lack of a placebo or control group [6], performed on participants not meeting the minimum age criteria (<16 years), and [7] the combination of beta-alanine with other supplements when compared with a placebo group.

## 6. Data extraction

Two independent investigators (DAL and OA) completed screening studies and data extraction from each qualified study. Extracted data contained the name of the primary investigator, year of publication, country of origin, study design, participant group size (placebo/control and intervention), participant demographics [(mean ± standard deviation [SD], age, body mass index (BMI), and sex)], beta-alanine dosage, duration of intervention, mean ± SD of body composition changes for both intervention and control groups, and any confounding variables utilized or accounted for in the randomized controlled trial (RCT). Dataset values were converted to the most common units of expression, whenever possible, for data analysis purposes.

## 7. Quality assessment

Study quality was measured by two independent reviewers (DAL and OA) using the Cochrane Collaboration modified risk of bias tool, which determines study bias in seven domains, including random sequence generation, allocation concealment, reporting bias, performance bias, detection bias, attrition bias, and other potential sources of bias [16]. Consequently, terms including ‘Low’, ‘High’, or ‘Unclear’ were used to classify each domain of study bias. Dissimilarities between independent reviewers on the level of study bias in each domain were evaluated and resolved by the corresponding author.

## 8. Statistical analysis

Weighted mean differences (WMD) and SDs of body composition (body mass, FM, BFP, and FFM) from both intervention and control groups were extracted and used to generate overall effect sizes as determined by the random-effects model approach of DerSimonian and Laird [17]. Additionally, when mean changes were not reported following beta-alanine supplementation (i.e. only mean value at baseline and again at post-intervention were noted in the study), the following formula was used to derive such changes: mean change = final post-intervention body composition indices value − baseline value for the same; and subsequently, changes in SDs of mean change scores were calculated by the following formula [18]:

$$SD\;change=\sqrt{[\left(SD\;baseline\right){\;}^{\wedge }2\;+\;\left(SD\;final\right){\;}^{\wedge }2\;-\;\left(2R\times SD\;baseline\times SD\;final\right)}.$$

The correlation coefficient (R) was considered as 0.8 (between 0 and 1), which is in accordance with prior meta-analytic work [18–20]. Moreover, reported standard errors (SEs), 95% confidence intervals (CIs), and interquartile ranges (IQRs) were converted to SDs using the method of Hozo et al. [21]. Subsequently, a random-effects model, which incorporates between-study variations, was utilized to determine the overall body composition effect size. Heterogeneity between studies was performed using Cochran’s Q test and analyzed by an I-square (I^2^) statistic [22] where I^2^ > 40% or *p* < 0.01 was considered as having high between-study heterogeneity [23]. Sensitivity analysis was undertaken to determine the individual study effect on the overall estimation of effect [24]. The possibility of publication bias was further verified through Begg’s test and funnel plots [25]. STATA, version 11.2 (Stata Corp, College Station, TX), was used to perform statistical analysis. *P*-values <0.05 were considered statistically significant for all analyses.

## 9. Certainty assessment

The overall certainty of evidence across studies was assessed based on GRADE (Grading of Recommendations Assessment, Development, and Evaluation) guidelines working group (gradeworkinggroup.org) [26]. The quality of evidence was subsequently classified into four categories according to the corresponding evaluation criteria, including high, moderate, low, and very low [27].

## 10. Results

### Study selection

**Figure 1.**
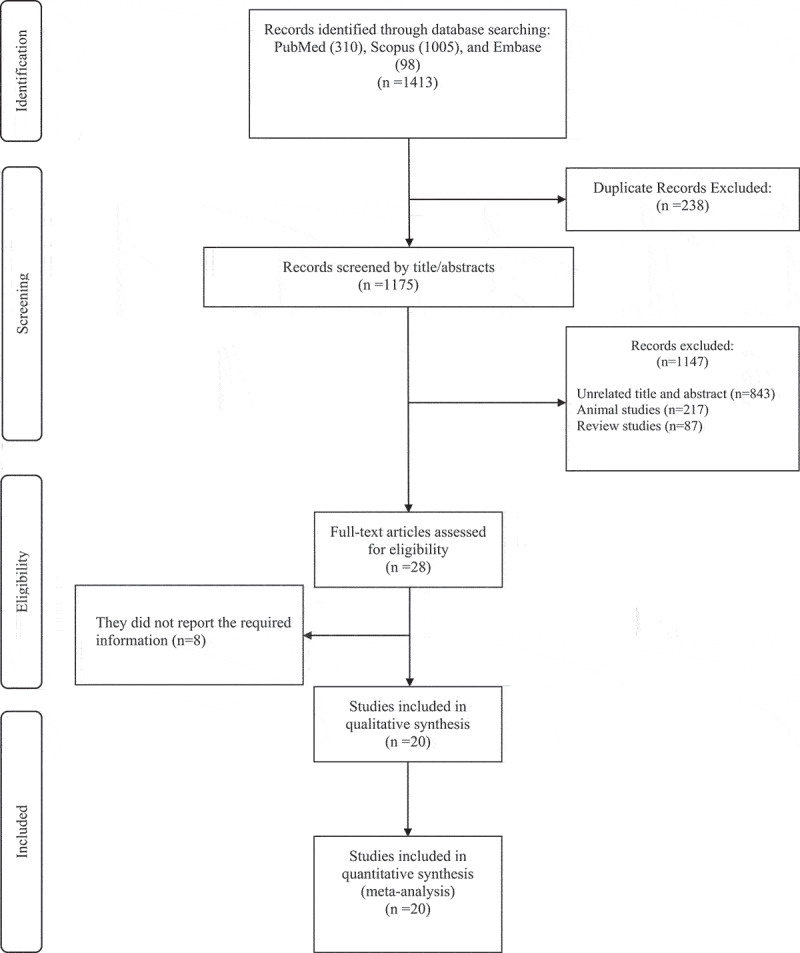
Flowchart of study selection for inclusion trials in the systematic review.

The initial databases search yielded 1413 studies, 238 of which were removed due to duplication. Another 1147 studies were excluded for the following reasoning: unrelated title and abstract not warranting full-text review (n = 843), animal (n = 217) and review studies (n = 87). Consequently, 28 relevant studies remained for full-text review and meta-analysis consideration. Eight studies were excluded because of a lack of necessary data reporting or other required information as outlined in the inclusion/exclusion criteria. Finally, 20 studies achieving all necessary criteria were included for meta-analysis in the present study (Figure 1).

### Study characteristics

The 20 included studies [11–14,28–43] contained a total of 25 intervention arms, which are shown in Table 1. These studies were published between 2008 and 2021, and in total, 492 participants were included. The study design of 19 studies was parallel (case = 242 participants and control = 242 participants), and one study had a crossover design (8 participants). Study duration varied from 3 to 10 weeks, while sample sizes ranged from 8 to 36 participants. Participants’ ages ranged from 17.4 to 53.5 years and baseline BFP from 7.8% to 35.7%. Beta-alanine dosage range was between 1.6 and 6.4 g/d. Except for two studies [31,39], others used beta-alanine supplementation combined with exercise training. Furthermore, most investigations (13 studies) were performed on men, whereas four studies utilized women and three included participants of both sexes. Quality assessment characteristics of studies are provided in Table 2.

**Table 1.** Characteristics of included studies in the meta-analysis

Study	Participants	Study design	Exercise intervention	Sample size (intervention/control)	Duration	Body analyzer method	Mean age	Mean BFP	Beta-alanine group	Control group	Effects of beta-alanine on body mass*	Effects of beta-alanine on BFP*	Effects of beta-alanine on FFM*
Shbib et al. 2021	Male handball players	RA/DB/PC (Parallel)	RT. Four exercises of 3 sets of 12 reps were applied in this plyometric regimen were added to the regular handball practice, 3 sessions/wk	18 (9/9)	4 wk	BIA	21.6	15.7	0.1 g/kg/d beta-alanine	0.1 g/kg/d maltodextrin	↔	↔	ND
Delextrat et al. 2020	Amateur male and female team- and racket sport players	RA/DB/PC (Parallel)	CT. 5-h of weekly training, including 2-h of RT and 3-h of aerobic-based exercise mixed with tactical work.	21 (10/11)	4 wk	BIA	24.7	16.3	6 g/d beta-alanine	11 g/d rice flour	↔	↔	ND
				23 (12/11)	4 wk	BIA	25.8	14.7	6 g/day beta-alanine + 5 g/d creatine	5 g/d creatine	↓	↔	ND
Smith et al. 2019	Male collegiate rugby players	RA/DB/PC (Parallel)	CT. Participants were engaged in weekly team-based strength and conditioning sessions and weekly team practices focusing upon strategy and conditioning.	15 (8/7)	6 wk	DXA	21	21.3	6.4 g/d beta-alanine	6.4 g/d maltodextrin	↔	↔	↔
Hooshmand et al. 2019	Sedentary overweight women	RA/DB/PC (Parallel)	No exercise intervention. Participants were asked to continue their routine physical activity	34 (17/17)	6 wk	BIA	20-45	35.7	1.6 g/d beta-alanine	Placebo tablets	↔	↔	↔
Freitas et al. 2019	Recreationally resistance-trained men	RA/DB/PC (Parallel)	RT. The program consisted of 5-7 exercises, three sets of 10-12 RM with 90-120 s of rest between sets.	22 (11/11)	4 wk	BIA	23.7	18.1	6.4 g/d beta-alanine	6.4 g/d maltodextrin	↔	↔	↔
Jaques et al. 2019	Male and female rowers	RA/DB/PC (Parallel)	CT. 6 sessions/week for two hours (a combination of a rowing specific endurance work and rowing drills to refine rowing technique) and two, one-hour RT sessions.	22 (10/12)	4 wk	Bod Pod	19.3	20.4	3.2 g/d beta-alanine (with powdered lemonade mix)	Powdered lemonade mix	↔	↔	↔
Askari et al. 2019	Resistance-trained men	RA/PC (Parallel)	RT. The program consisted of 8 exercises of all major muscle groups, three sets of 8-12 RM with 70-80% of 1RM, 3 sessions/week, and 85 min/session.	20 (10/10)	8 wk	Skinfold thickness measurement	17.4	14.1	4.8 g/d beta-alanine	4.8 g/d polydextrose	↔	↔	ND
Jaffe et al. 2018	Physically active males	RA/DB/PC (Parallel)	Moderate to maximal-effort total body weight lifting, sprinting, plyometric exercises, and regular endurance training	30 (16/14)	6 wk	Bod Pod	20.5	15.3	6 g/d beta-alanine	6 g/d maltodextrin	ND	↔	↔
Wang et al. 2018	Recreationally active men	RA/DB/PC (Parallel)	CT. 5–7 hours of resistance or endurance training per week (8 training sessions over 4 weeks) in normoxia or hypoxia	19 (11/8)	4 wk	Bod Pod	22.6	19.4	6.4 g/d beta-alanine	6.4 g/d rice powder	↔	↔	ND
				19 (10/9)	4 wk	Bod Pod	22.6	15	6.4 g/d beta-alanine	6.4 g/d rice powder	↔	↔	ND
Glenn et al. 2016	female masters cyclists	RA/DB/PC (Parallel)	ET. Details were ND .	22 (11/11)	4 wk	DXA	53.5	30.5	3.2 g/d beta-alanine + 32 g dextrose	32 g dextrose	ND	↔	ND
Gross et al. 2014	professional alpine skiers	DB/PC (Parallel)	CT. it consists of high volumes of strength and conditioning training and on-snow ski training.	9 (5/4)	5 wk	Skinfold thickness measurement	19.5	12.7	4.8 g/d beta-alanine	4.8 g/d maltodextrin	↔	↔	ND
Kresta et al. 2014	Recreationally active female	RA/DB/PC (Parallel)	CT. exercise such as running, cycling, swimming, resistance training, fitness classes for at least 30 minutes per day for 3-days per-week	15 (8/7)	4 wk	DXA	21.5	27.8	0.1 g/kg/d beta-alanine	0.1 g/kg/d maltodextrin	↔	↔	↔
				17 (9/8)	4 wk	DXA	21.5	25.6	0.1 g/kg/d beta-alanine + 0.3 g/kg/d of creatine for week 1 and 0.1 g/kg/day for weeks 2–4.	0.3 g/kg/d of creatine for week 1 and 0.1 g/kg/day for weeks 2–4.	↔	↔	↔
Hoffman et al. 2014.	Male combat soldiers	RA/DB/PC (Parallel)	CT. It consists of military training tasks, including combat skill development, physical work under pressure, navigational training, self-defense/ hand-to-hand combat, and conditioning.	18 (9/9)	4 wk	ND	20.1	ND	6 g/d beta-alanine	6 g/d rice powder	↓	ND	ND
Sale et al. 2012	physically active males	PC (Parallel)	ND (participants were requested to maintain similar levels of physical activity)	13 (7/6)	4 wk	ND	23	ND	6.4 g/d beta-alanine	6.4 g/d maltodextrin	↔	ND	ND
Outlaw et al. 2012	Untrained collegiate females	RA/DB/PC (Parallel)	RT. Four-day-per-week RT program using an upper and lower-body split program at ~65% of 1RM.	15 (7/8)	8 wk	DXA	21	30.1	3.4 g/d beta-alanine	5 g/d maltodextrin	↔	↔	↔
Kern et al. 2011	Collegiate wrestlers and football players	RA/DB/PC (Parallel)	CT. wrestlers participated in 4–5 d/week practice sessions (HIIT) and 3 d/weeks RT. Football players practiced 3 d/week and participated in RT sessions 4 d/week.	15 (7/8)	8 wk	Skinfold thickness measurement	18.6	9.67	4 g/d beta-alanine	4 g/d placebo (in powdered capsule)	↔	↔	↔
				22 (10/12)	8 wk	Skinfold thickness measurement	19.9	7.8	4 g/d beta-alanine	4 g/d placebo (in powdered capsule)	↔	↔	↔
Walter et al. 2010	Recreationally active female	RA/DB/PC (Parallel)	ET. High intensity interval training on 3 nonconsecutive days per week.	33 (14/19)	3 wk	Bod Pod	21.6	30	6 g/d beta-alanine + 60 g dextrose	66 g dextrose	↑	↔	↔
Smith et al. 2009	Recreationally active male	RA/DB/PC (Parallel)	ET. High-intensity interval training which first three-week period of training was completed at workloads between 90%–110% of each individual’s VO2peak, while the second three-week training peaked at 115%.	36 (18/18)	3 wk	Bod Pod	22.2	14.9	6 g/d beta-alanine + 60 g dextrose	66 g dextrose	↔	↔	↑
Kendrick et al. 2008	Physical education male student	DB/PC (Parallel)	RT. 4 days/week for 10 weeks. Two sessions per week were upper body dominant, and two were lower body dominant	26 (13/13)	10 wk	Skinfold thickness measurement	21.5	10.1	6.4 g/d beta-alanine	6.4 g/d maltodextrin	↔	↔	ND
Hoffman et al. 2008	Resistance-trained male	DB/PC (cross-over)	RT. The program consisted of 9 exercises, 8-10 of 1-RM with 1.5-2 min of rest between sets, 4 sessions/week.	8 (8/8)	4 wk	ND	19.7	15.7	4.8 g/d beta-alanine	4.8 g/d placebo	↔	ND	ND

**Abbreviations**. 1-RM, 1-repetition maximum; BFP, body fat percentage; BIA, Bioelectrical impedance analysis; CO, controlled; CT, combined training; d, day; DB, double-blinded; DEXA, dual-energy x-ray absorptiometry; ET, endurance training; FFM, fat-free mass; HIIT, high-intensity interval training; kg, kilogram; ND, non-defined; PC, placebo-controlled; RA, randomized; RT, resistance training; SB, single-blinded. *Compared to placebo group.

**Table 2.** Quality assessment

Studies	Random sequence generation	Allocation concealment	Selective reporting	Other sources of bias	Blinding (participants and personnel)	Blinding (outcome assessmen)	Incomplete outcome data	Overall quality
Shbib et al. 2021	U	U	H	H	L	L	L	High-risk
Delextrat et al. 2020	U	H	H	H	L	L	L	High-risk
Smith et al. 2019	U	U	L	H	L	L	L	High-risk
Hooshmand et al. 2019	L	L	L	H	L	L	L	Moderate-risk
Freitas et al. 2019	U	H	L	H	L	L	L	High-risk
Jaques et al. 2019	L	L	H	H	L	L	L	High-risk
Askari et al. 2019	U	L	H	H	H	H	L	High-risk
Jaffe et al. 2018	U	H	H	H	L	L	L	High-risk
Wang et al. 2018	L	H	H	H	H	L	L	High-risk
Glenn et al. 2016	U	L	H	H	L	L	L	High-risk
Gross et al. 2014	H	H	H	H	L	L	L	High-risk
Kresta et al. 2014	U	L	L	H	L	L	L	Moderate-risk
Hoffman et al. 2014.	L	U	H	H	L	L	L	High-risk
Sale et al. 2012	H	U	H	H	H	H	L	High-risk
Outlaw et al. 2012	U	L	H	H	L	L	H	High-risk
Kern et al. 2011	U	H	H	H	L	L	L	High-risk
Walter et al. 2010	U	L	H	H	L	L	L	High-risk
Smith et al. 2009	U	L	L	H	L	L	L	Moderate-risk
Kendrick et al. 2008	H	L	H	H	L	L	H	High-risk
Hoffman et al. 2008	U	L	H	H	L	L	L	High-risk

**Abbreviations**. H, high; L, low; U, unclear.

## 11. Meta-analysis

### The effects of beta-alanine supplementation on body mass

Outcomes analysis of the 16 studies (21 arms in total) [11,12,14,28–33,36–38,40–43] (n = 387) that measured body mass following beta-alanine supplementation did not show an overall effect of a significant change in body mass (WMD: −0.15 kg; 95% CI: −0.78 to 0.47; *p* = 0.631, I^2^ = 0.0%, *p* = 0.998) (Figure 2(a)). In addition, all subgroup analyses did not indicate any changes in body mass following beta-alanine supplementation (Table 3).

**Table 3.** Subgroup analyses of beta-alanine supplementation on body composition

				heterogeneity			
	NO	WMD (95% CI)	P	P heterogeneity	I^2^	P between sub-groups	Tau-squared
Subgroup analyses of beta-alanine supplementation on body mass							
Overall effect	21	−0.15 (−0.78, 0.47)	0.631	0.998	0.0%		0.0
Exercise type							
RT	4	−0.09 (−1.03, 0.85)	0.851	0.829	0.0%	0.856	0.0
ET	3	0.75 (−2.45, 3.96)	0.644	0.984	0.0%		0.0
CT	13	−0.15 (−1.11, 0.81)	0.758	0.961	0.0%		0.0
Duration (week)							
<8	18	−0.25 (−1.23, 0.71)	0.602	0.999	0.0%	0.783	0.0
≥8	3	−0.07 (−0.90, 0.74)	0.852	0.357	2.9%		0.02
Dose (g/d)							
<6	7	−0.11 (−1.24, −1.24)	0.846	0.655	0.0%	0.931	0.0
≥6	14	−0.17 (−0.93, 0.58)	0.655	0.999	0.0%		0.0
Subgroup analyses of beta-alanine supplementation on fat mass							
Overall effect	7	−0.24 (−1.16, 0.68)	0.612	0.969	0.0%		
Exercise type							
RT	2	0.34 (−2.51, 3.21)	0.813	0.874	0.0%	0.802	0.0
ET	1	0.10 (−2.86, 3.06)	0.947	-	-		0.0
CT	3	0.24 (−1.48, 1.97)	0.781	0.853	0.0%		0.0
Duration (week)							
<8	6	−0.29 (−1.26, 0.66)	0.544	0.950	0.0%	0.662	0.0
≥8	1	0.50 (−2.94, 3.94)	0.776	-	-		0.0
Dose (g/d)							
<6	2	−0.55 (−1.76, 0.66)	0.375	0.523	0.0%	0.439	0.0
≥6	5	0.19 (−1.24, 1.62)	0.793	0.988	0.0%		0.0
Subgroup analyses of beta-alanine supplementation on body fat percentage							
Overall effect	21	−0.06 (−0.53, 0.40)	0.782	0.936	0.0%		0.0
Exercise type							
RT	4	0.19 (−1.51, 1.90)	0.823	0.564	0.0%	0.717	0.0
ET	2	0.02 (−1.87, 1.91)	0.983	0.879	0.0%		0.0
CT	13	0.05 (−0.51, 0.62)	0.849	0.801	0.0%		0.0
Duration (week)							
<8	17	−0.11 (−0.74, 0.51)	0.729	0.901	0.0%	0.832	0.0
≥8	4	−0.01 (−0.70, 0.69)	0.978	0.566	0.0%		0.0
Dose (g/d)							
<6	9	−0.16 (−0.70, 0.37)	0.546	0.513	0.0%	0.464	0.0
≥6	12	0.23 (−0.70, 1.18)	0.618	0.980	0.0%		0.0
Subgroup analyses of beta-alanine supplementation on fat-free mass							
Overall effect	13	0.05 (−0.71, 0.82)	0.889	0.912	0.0%		0.0
Exercise type							
RT	2	0.06 (−2.12, 2.25)	0.951	0.581	0.0%	0.684	0.0
ET	2	1.25 (−1.64, 4.14)	0.396	0.408	0.0%		0.0
CT	8	0.31 (−0.87, 1.50)	0.600	0.825	0.0%		0.0
Duration (week)							
<8	10	−0.13 (−0.99, 0.73)	0.764	0.921	0.0%	0.357	0.0
≥8	3	0.75 (−0.91, 2.41)	0.378	0.504	0.0%		0.0
Dose (g/d)							
<6	6	−0.08 (−1.03, 0.87)	0.866	0.722	0.0%	0.637	0.0
≥6	7	0.30 (−0.98, 1.59)	0.644	0.810	0.0%		0.0

**Abbreviations**. CI, confidence interval; WMD, weighted mean differences; RT, resistance training; ET, endurance training; CT, combined training;

**Figure 2.**
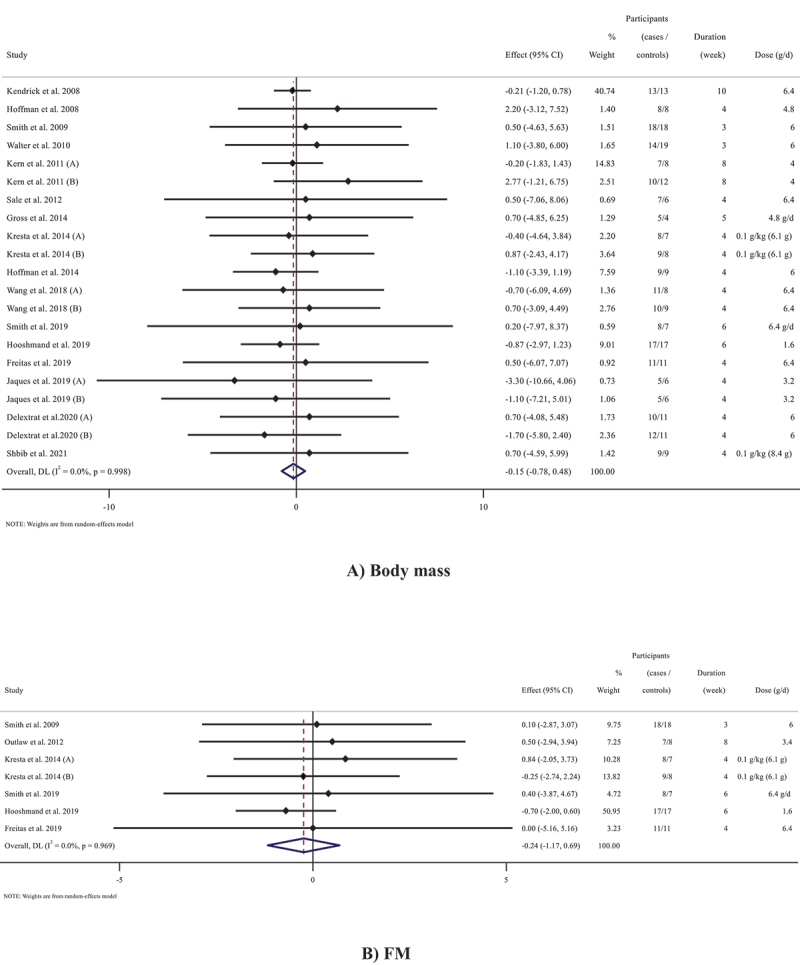
Forest plot detailing weighted mean difference and 95% confidence intervals (CIs) for the effect of beta-alanine supplementation on A) body mass; B) FM; C) BFP; D) FFM.

**Figure 2.**
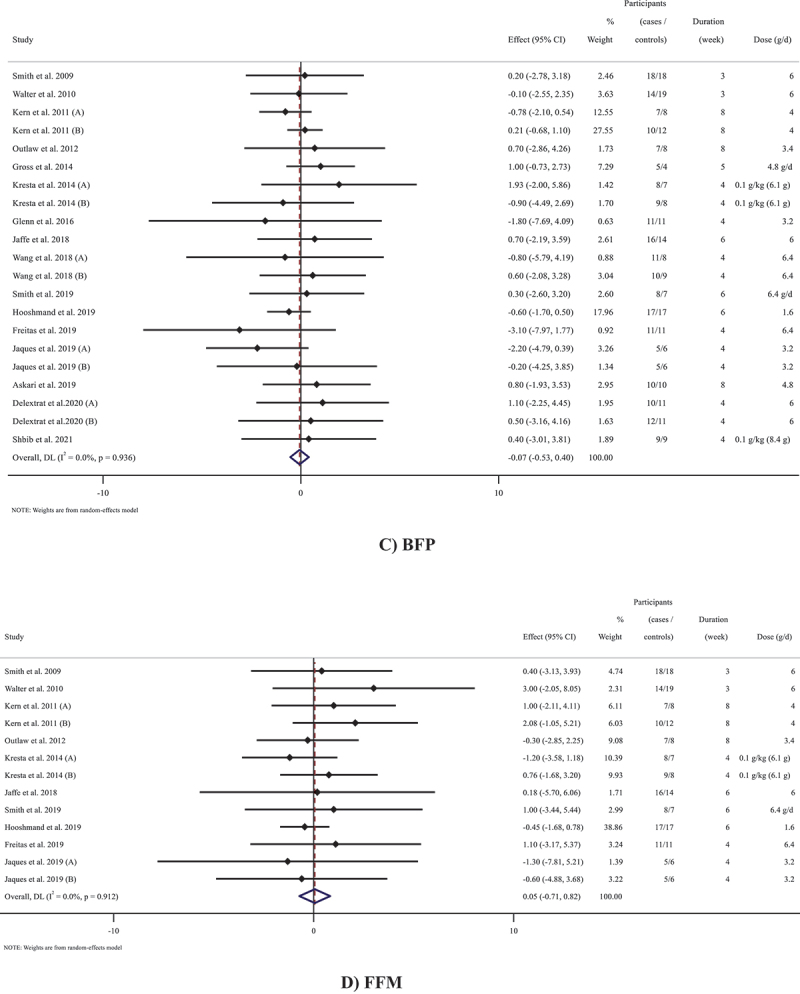
(Continued).

### The effects of beta-alanine supplementation on FM

Based on the results of six studies [11,30–32,39,43] containing 7 total effect sizes (n = 154), beta-alanine supplementation failed to change FM (WMD: −0.24 kg; 95% CI: −1.16 to 0.68; *p* = 0.612, I^2^ = 0.0%, *p* = 0.969) (Figure 2(b)) regardless of exercise type, study duration, and the dose of supplementation (Table 3).

### The effects of beta-alanine supplementation on BFP

Overall result from 16 studies [11,13,28–39,43] containing 21 total effect sizes (n = 427) did not reveal significant alterations in BFP (WMD: −0.06%; 95% CI: −0.53 to 0.40; *p* = 0.782, I^2^ = 0.0%, *p* = 0.936) (Figure 2(c)). Insignificant changes were shown in all subgroups (Table 3).

### The effects of beta-alanine supplementation on FFM

Pooled effect sizes from 10 studies [11,30–33,35,37–39,43] containing 13 arms (n = 276) did not reveal a significant change in FFM following beta-alanine supplementation (WMD: 0.05 kg; 95% CI: −0.71 to 0.82; *p* = 0.889, I^2^ = 0.0%, *p* = 0.912). Subgroup analyses demonstrated similar results (Figure 2(d) and Table 3).

### Publication bias

**Figure 3.**
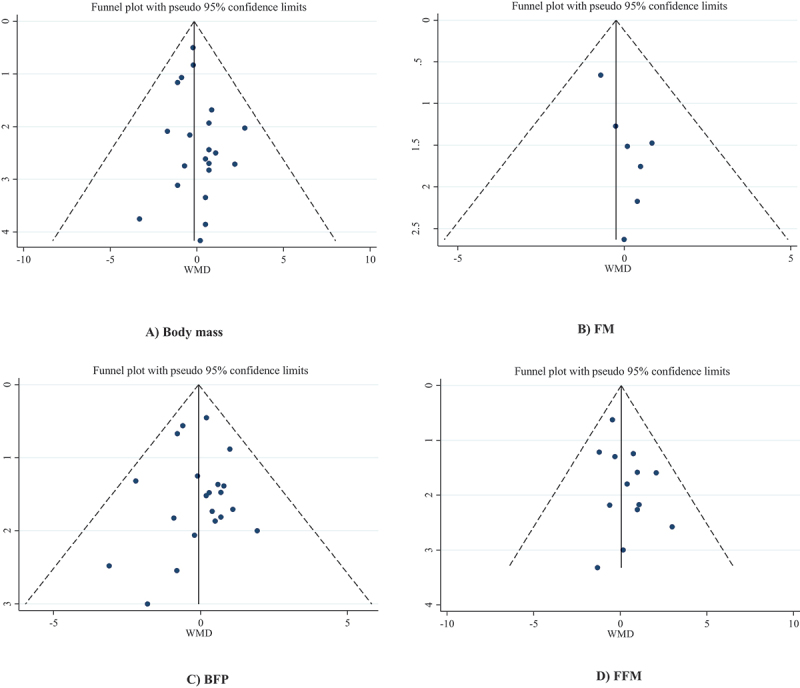
Funnel plot for the effect of beta-alanine supplementation on (A) body mass; (B) FM; (C) BFP; (D) FFM.

According to Begg’s regression test, there was no evidence of publication bias for studies examining the effect of beta-alanine supplementation on body mass (*p* = 0.786), FM (*p* = 0.548), BFP (*p* = 0.349), and FFM (*p* = 0.760). In addition, Egger’s regression test showed no significant publication bias for body mass (*p* = 0.285), BFP (*p* = 0.881), and FFM (*p* = 0.110), but there was evidence of publication bias found for FM (*p* = 0.031). The trim and fill analysis for FM demonstrated that, with the addition of 11 unpublished articles, the test for publication bias was no longer significant; however, the overall effect did not change significantly (WMD: −0.575, 95%CI: −1.382 to 0.232; *p* = 0.162). Funnel plots indicated no evidence of asymmetry in the effects of beta-alanine supplementation on all body composition indices except for FM (Figure 3(a–d)).

### Sensitivity analysis

Upon removing individual study effects for sensitivity analysis, the overall results did not significantly change for body mass, FM, BFP, and FFM.

### Grading of evidence

The GRADE protocol was used to evaluate the certainty of the evidence (Table 4). The quality of evidence related to body mass, FM, and BFP was downgraded to moderate due to serious limitations in risk of bias. Moreover, the GRADE assessment for FFM was low due to concerns about both risk and publication bias.

**Table 4.** GRADE profile of beta-alanine supplementation on body composition

Quality assessment							
Outcomes	Risk of bias	Inconsistency	Indirectness	Imprecision	Publication Bias	Sample sizes (cases/control)	Quality of evidence
Body mass	Serious Limitations ^a^	No Serious Limitations	No Serious Limitations	Serious Limitations ^b^	No serious limitations	413 (206/207)	⊕⊕◯ ◯ Low
Fat Mass	Serious Limitations ^a^	No Serious Limitations	No Serious Limitations	Serious Limitations ^b,c^	No serious limitations	154 (78/76)	⊕⊕◯ ◯ Low
Body Fat Percentage	Serious Limitations ^a^	No Serious Limitations	No Serious Limitations	Serious Limitations ^b^	No serious limitations	427 (213/2014)	⊕⊕◯ ◯ Low
Fat-free mass	Serious Limitations ^a^	No Serious Limitations	No Serious Limitations	Serious Limitations ^b^	Serious Limitations ^d^	254 (125/129)	⊕◯ ◯ ◯ Very Low

^a^most of the studies have high-risk of bias

^b^failed to meet significant effect (CI includes WMD of ‘0’)

^c^due to small sample sizes (n < 198)

^d^there is publication bias for FM (*p* = 0.031).

## 12. Discussion

The purpose of this study was to determine if beta-alanine supplementation in doses of 1.6-8.4 g/d improves body composition indices. Overall, beta-alanine supplementation exerted no significant impact on body mass, FM, BFP, and FFM. Subgroup analysis based on dosage (*≥*6 g or <6 g/d), study duration (8 ≥ or 8 < weeks), and exercise type (resistance, endurance, and combined training) indicated no significant changes following beta-alanine supplementation on body composition.

To our knowledge, this is the first systematic review and meta-analysis investigating the longitudinal effects of beta-alanine supplementation on body composition indices. Although its role as a precursor to the dipeptide carnosine has led recent researchers to consider beta-alanine as an ergogenic aid to improve exercise performance, some investigations have failed to show any significant improvements in exercise performance variables such as strength, endurance, and power following beta-alanine supplementation [44]. The contribution of carnosine to intracellular buffering during intense exercise can attenuate intracellular acidosis as a possible factor contributing to reduced exercise performance [45]. Other putative physiological effects of carnosine, such as increased calcium sensitivity [46] and antioxidant capabilities [47], may also have a positive impact on exercise performance, but the data is equivocal [48]. However, most studies did not show any improvements in body composition indices following beta-alanine supplementation, indicating that the beneficial effects of intramuscular carnosine accumulation did not translate into body composition changes [14,39,42,49].

Pooled analysis of the studies included in this meta-analysis found no significant changes in body mass or FFM following beta-alanine supplementation. The results from our study were in line with previous RCTs, which did not observe any positive effects of beta-alanine supplementation on FFM [11,14,35,37,40]. In this regard, in college-aged women, Outlaw et al. showed that beta-alanine supplementation (3.4 g/day) for 8 weeks combined with resistance training increased lower-body muscular endurance but had no effect on maximal strength, FFM, FM, or BFP [39]. In addition, Kresta et al. assessed the influences of beta-alanine and creatine supplementation on muscle carnosine, body composition, and exercise performance in recreationally active females over 28 days and reported no FFM improvements [43].

Although beta-alanine supplementation appears to be a valuable ergogenic aid in HIIT requiring a high degree of strength endurance, its capacity to boost hypertrophic responses during resistance training remains unknown. The observed beneficial effects of beta-alanine supplementation on lean mass in prior research can be attributable to beta-alanine’s ability to promote fluid shifts into muscle and subsequent increases in intramuscular water, which have been claimed to account for part of the gains in FFM [12,50]. However, Freitas et al. showed that 28 days of beta-alanine supplementation did not increase total or intracellular water content during resistance training after HIIT [32]. In addition, recent studies were unable to measure intramuscular carnosine concentrations or myofibrillar protein content in the exercised muscles to prove their claims toward beneficial effects of beta-alanine supplementation on lean mass through muscle hypertrophy [32,40,41]. As can be seen from the subgroup analysis, differences between studies in terms of study duration, dosages of beta-alanine, and exercise type did not change the overall impacts of beta-alanine supplementation on body mass or lean mass. It should be mentioned that only two of the 13 included studies on the effects of beta-alanine supplementation on FFM lasted 8 weeks [12,39]. The findings of a previous review implied that the supplementation time might be a modifying factor for the ergogenic effect of beta-alanine [51]. It is possible that shorter supplementation procedures (e.g. 3 weeks) are insufficient to meet the threshold of muscle carnosine level increases required to improve Yo-Yo test performance [51]. In this regard, one study found that beta-alanine supplementation and placebo treatment were equally effective at improving VO2peak, time to fatigue, and total work performed over 3 weeks of HIIT in young men [11]; however, only the group that supplemented with beta-alanine showed an increase in total work performed and lean mass after 6 weeks of training [11], supporting this theory. The usage of beta-alanine has been suggested by scientists as a method to improve training adaptation by enhancing the ability to train at a higher intensity with less muscle fatigue [52,53]. Future studies are needed with longer-term beta-alanine supplementation duration with HIIT or resistance training on alterations in FFM.

We also did not find significant alterations following beta-alanine supplementation on FM or BFP. Previous RCTs showed that both acute and chronic supplementation with beta-alanine in different practical settings resulted in small and non-significant effects on FM or BFP [13,33,43,54]. For example, Smith et al. found that 6 weeks of beta-alanine supplementation combined with HIIT was failed to significantly change FM in recreationally active men [11]. Moreover, a recent study examined 6 weeks of beta-alanine supplementation in overweight women and reported increased time to exhaustion on a treadmill test compared to the control group, while FM remained unchanged [31]. In a study conducted by Hoffman et al., a significant decrease in BFP was reported in the beta-alanine plus creatine supplementation group compared to the control but not different from the creatine supplementation group only [42]. The potential benefit of adding beta-alanine with creatine for the 10-week duration may increase the fatigue resistance, allowing for greater training volume [55]. Hoffman et al. reported no significant difference in total kcals between groups; thus, greater weekly training volume in the beta-alanine and creatine group vs. the placebo group would result in more significant kcal expenditure, thus potentially having a secondary effect on FM.

What remains to be seen is why the potential effects of beta-alanine supplementation on intramuscular carnosine concentrations and its contribution to intracellular buffering have yet to be translated into the improvements in body composition consistently. It should be noted that this lack of improvement could be due to some methodological and participant-related criteria in the previous studies. First of all, the primary aim of the majority of these studies was to increase exercise performance, and consequently, their training interventions were not specifically designed for achieving and maximizing body composition alterations. Second, it is important to note that most previous studies employed well-trained men with a lower baseline BFP; thus, the further fat loss was unexpected. Finally, the positive effects of beta-alanine supplementation on improvements in exercise performance are thought to be due to increases in intramuscular concentrations of carnosine, although the majority of RCTs included in our investigation did not measure carnosine concentrations [11,31,32,35–38,40,41,54]. Therefore, the results of these studies should be interpreted with caution. It is worth mentioning that the increases in intramuscular concentrations of carnosine appear to be influenced by baseline levels and habitual dietary intake of carnosine-containing foods, which can potentially affect the impacts of beta-alanine supplementation on exercise performance and thereby body composition changes [56,57].

The current meta-analysis has some limitations. As mentioned above, in the majority of included RCTs, baseline intracellular carnosine concentrations, its changes during the study period, or dietary intake of carnosine and total protein using a validated methodology, such as 24-hour food recalls, were not investigated. Another significant limitation of this meta-analysis was the scarcity of research that used body composition indices as a primary outcome. However, low heterogeneity across the included studies’ results must be considered a strength of this study. Moreover, according to the GRADE profile, the quality of evidence for FFM is low. In other words, our confidence in the FFM-enhancing properties of beta-alanine supplementation is limited. However, in terms of quality of evidence of the effects of beta-alanine supplementation on body mass, BFP and FM, we are moderately confident in the effect estimate.

## 13. Conclusions

Beta-alanine supplementation does not improve body composition, and subgroup analysis based on study duration, beta-alanine dosage, and different training types did not alter the observed results. Because all studies included in the present systematic review and meta-analysis lasted less than 3 months, additional longer-term RCTs are necessary to expand our findings.

## Data Availability

Data sharing is applicable.

## Authors’ information

This was provided in the first page.
